# Supplementary material for: A New Molecular Detection System for Canine Distemper Virus Based on a Double-Check Strategy
Source: Viruses. 2021 Aug 18;13(8):1632. doi: 10.3390/v13081632 (PMC8402888; doi:10.3390/v13081632)
Supplement: Supplementary file 1 [file viruses-13-01632-s001.zip › viruses-1309197-supplementary.pdf]

viruses MDPI

Supplementary material

A New Molecular Detection System for Canine Distemper Virus Based on a Double-Check Strategy

Sabrina Halecker[1,†], Sabine Bock[2], Martin Beer[1], Bernd Hoffmann[1*]

[1]Institute of Diagnostic Virology, Friedrich-Loeffler-Institut, Federal Research Institute for Animal Health, Südufer 10, 17493 Greifswald-Insel Riems, Germany
[2] Landeslabor Berlin-Brandenburg, Gerhard-Neumann-Str. 2, 15236 Frankfurt (Oder), Germany.
†current adress: Institute of Virology, Freie Universität Berlin, Robert von Ostertag-Straße 7-13, 14163 Berlin, Germany
* Correspondence: Bernd.Hoffmann@fli.de, Tel.: +49 38351 7 1506

Table S1 Comparative test series on initial 13 CDV RT-qPCR assays carried out with AgPath-ID™ One-Step RT-PCR Reagents and based on a log10 dilutional series (10^{-1} to 10^{-8}) of the CDV strain "Onderstepoort" (sample ID: BH 04/18-2) by contrast with the ELIA-Mix, location within the genome are shown for each single CDV RT-qPCR assay, results are shown in Cq values

location:	**N gene**	**P gene**									**L gene**			
	**ELIA-Mix**	**CDV-Mix 3**	**CDV-Mix 4**	**CDV-Mix 5**	**CDV-Mix 6**	**CDV-Mix 7**	**CDV-Mix 8**	**CDV-Mix 9**	**CDV-Mix 10**	**CDV-Mix 11**	**CDV-Mix 12**	**CDV-Mix 13**	**CDV-Mix 14**	**CDV-Mix 15**
**10^{-1}**	21.4	19.7	19.6	19.2	19.1	18.6	19.4	19.5	19.2	19.6	20.1	19.8	19.2	18.9
**10^{-2}**	24.3	23.0	23.1	22.4	22.5	22.1	22.7	23.0	22.5	22.6	23.4	23.1	22.5	22.3
**10^{-3}**	28.0	26.4	26.5	25.7	26.0	25.2	26.1	26.2	25.7	26.0	26.3	26.5	25.8	25.7
**10^{-4}**	31.6	29.7	30.0	29.1	29.3	28.7	29.4	29.6	29.1	29.1	29.4	30.1	29.2	29.1
**10^{-5}**	35.8	33.0	33.5	32.7	32.6	32.2	32.6	33.2	No Cq	35.1	34.5	33.3	32.7	32.2
**10^{-6}**	39.3	35.0	36.1	36.8	35.5	35.0	37.5	36.4	No Cq	No Cq	No Cq	39.2	34.9	36.5
**10^{-7}**	No Cq	No Cq	No Cq	No Cq	No Cq	No Cq	No Cq	39.0	No Cq	No Cq	No Cq	No Cq	38.2	38.2
**10^{-8}**	No Cq	No Cq	No Cq	No Cq	No Cq	No Cq	No Cq	No Cq	No Cq	No Cq	No Cq	No Cq	No Cq	No Cq

RT-qPCR = reverse transcriptase quantitative polymerase chain reaction, CDV = canine distemper virus, ID = ident number; Cq = quantification cycler

Table S2 Comparative test series on initial 13 CDV RT-qPCR assays carried out on the Bio-Rad CFX96™ Real-Time PCR Detection System and based on a log10 dilutional series (10^{-1} to 10^{-8}) of the CDV strain "Onderstepoort" (sample ID: BH 04/18-2) by contrast with the ELIA-Mix, results are shown in RFU values

location:	**N gene**	**P gene**									**L gene**			
	**ELIA-Mix**	**CDV-Mix 3**	**CDV-Mix 4**	**CDV-Mix 5**	**CDV-Mix 6**	**CDV-Mix 7**	**CDV-Mix 8**	**CDV-Mix 9**	**CDV-Mix 10**	**CDV-Mix 11**	**CDV-Mix 12**	**CDV-Mix 13**	**CDV-Mix 14**	**CDV-Mix 15**
$\mathbf{10^{-1}}$	4955	4916	4404	5894	3914	6209	5731	6011	3282	3625	5749	6807	5836	6351
$\mathbf{10^{-2}}$	5179	5995	4429	6144	3830	6628	6040	6236	2994	3285	5080	6997	6354	6780
$\mathbf{10^{-3}}$	5216	5481	4211	6054	3815	6766	5886	6090	2201	2883	3943	7029	6238	6775
$\mathbf{10^{-4}}$	4960	5280	3734	6028	3171	6216	5561	6246	1138	1839	2434	6709	5794	6055
$\mathbf{10^{-5}}$	4093	4623	2534	5316	2367	5254	4530	5197	26	694	874	5772	4088	6074
$\mathbf{10^{-6}}$	2784	3924	1062	3897	1840	4105	2299	3562	-2	47	44	3882	3285	4678
$\mathbf{10^{-7}}$	28	11	16	16	22	15	9	3576	6	29	24	39	1468	4009
$\mathbf{10^{-8}}$	20	207	21	12	192	26	-5	13	26	12	7	8	23	24

RT-qPCR = reverse transcriptase quantitative polymerase chain reaction, CDV = canine distemper virus, ID = ident number; RFU = relative fluorescence unit

Table S3 Test series on the exclusivity and inclusivity of five preselected CDV RT-qPCR assays using different isolates of the four lineages of PPRV (L1 to L4), MV and *Cetacean morbilliviruses* (CeMV), test series carried out with isolates of representatives of paramyxovirues (NDV, Nipah virus, Hendra virus and BPIV-3) were negative for all five CDV-mixes (not shown here)

**CDV-Mix No.**	**PPRV**					**MV**		**PDV**		**CeMV**	
	**Ivory Coast/89 (L1)**	**Nigeria 75/1 (L2)**	**Sudan/72 (L3)**	**India/Shahjadpur (L4)**	**Kurdistan/2011 (L4)**	**MV Mor**	**MeV Anchorage**	**PDV**	**PDV**	**PMV**	**DMV**
**3**	No Cq	No Cq	No Cq	No Cq	No Cq	No Cq	No Cq	No Cq	No Cq	No Cq	No Cq
**5**	No Cq	No Cq	No Cq	No Cq	No Cq	No Cq	No Cq	No Cq	No Cq	No Cq	No Cq
**7**	No Cq	No Cq	No Cq	No Cq	No Cq	No Cq	No Cq	No Cq	No Cq	No Cq	No Cq
**9**	No Cq	No Cq	No Cq	No Cq	No Cq	No Cq	No Cq	35.1	31.0	No Cq	No Cq
**15**	36.0	33.7	25.1	27.1	31.2	No Cq	31.9	No Cq	No Cq	No Cq	34.5

CDV = canine distemper virus, RT-qPCR = reverse transcriptase quantitative polymerase chain reaction, PPRV = peste des petits ruminants virus, MV = measle virus, NDV = Newcastle disease virus, BPIV-3 = bovine parainfluenza virus 3, PDV = phocine distemper virus, PMV = porpoise mobillivirus, DMV = dolphin morbillivirus

Table S4 Validation data of positive samples carried out by the one-step RT-qPCR for CDV detection

Sample ID	Geographical origin		Year*	host	material	Cq values of RT-qPCR		
	Country	Federal state				CDV Mix 3	CDV Mix 7	ELIA Mix
BH 75/18-1A	Germany	Hesse	-	Fox	homogenate	24.4	23.8	22.3
BH 75/18-2A	Germany	Hesse	-	Fox	homogenate	29.4	29.2	27.2
BH 75/18-4A	Germany	Hesse	-	Fox	homogenate	26.7	26.0	27.2
BH 75/18-5A	Germany	Hesse	-	Fox	homogenate	28.2	28.1	29.6
BH 75/18-7A	Germany	Hesse	-	Fox	homogenate	30.0	29.2	31.7
BH 75/18-9A	Germany	Hesse	-	Fox	homogenate	21.8	22.5	21.1
BH 75/18-14A	Germany	Hesse	-	Fox	homogenate	24.6	24.9	25.9
BH 75/18-10A	Germany	Hesse	-	Fox	homogenate	21.6	21.9	19.3
BH 75/18-15A	Germany	Hesse	-	Fox	homogenate	20.1	20.7	18.5
BH 75/18-12A	Germany	Hesse	-	Fox	homogenate	27.1	27.1	26.2
BH 24/18-6	Germany	Lower Saxony	2016	Fox	organ grinding	30.0	29.3	27.3
BH 24/18-13	Germany	Berlin	2012	red fox	organ grinding	26.1	26.0	24.6
BH 75/18-1B	Germany	Hesse	-	Fox	brain	29.7	30.3	28.8
BH 75/18-2B	Germany	Hesse	-	Fox	brain	25.2	24.4	23.1
BH 75/18-9B	Germany	Hesse	-	Fox	brain	25.0	25.0	23.2
BH 75/18-14B	Germany	Hesse	-	Fox	brain	19.6	19.4	18.1
BH 75/18-10B	Germany	Hesse	-	Fox	brain	27.5	27.0	26.2
BH 75/18-15B	Germany	Hesse	-	Fox	brain	27.1	27.2	26.0
BH 75/18-12B	Germany	Hesse	-	Fox	brain	30.6	30.6	30.1
BH 24/18-2	Germany	Saxony-Anhalt		red fox	brain	26.1	25.3	24.6
BH 75/18-1C	Germany	Hesse	-	Fox	lung	22.2	23.5	20.2
BH 75/18-2C	Germany	Hesse	-	Fox	lung	30.1	31.0	28.6
BH 75/18-9C	Germany	Hesse	-	Fox	lung	21.6	23.3	20.1
BH 75/18-14C	Germany	Hesse	-	Fox	lung	24.2	25.7	22.6
BH 75/18-10C	Germany	Hesse	-	Fox	lung	24.8	25.2	22.4
BH 75/18-15C	Germany	Hesse	-	Fox	lung	22.8	22.8	20.0
BH 75/18-12C	Germany	Hesse	-	Fox	lung	25.8	26.7	24.0
BH 75/18-1D	Germany	Hesse	-	Fox	liver	25.1	26.5	23.7
BH 75/18-2D	Germany	Hesse	-	Fox	liver	36.2	36.1	34.1
BH 75/18-9D	Germany	Hesse	-	Fox	liver	25.3	25.8	23.3
BH 75/18-14D	Germany	Hesse	-	Fox	liver	26.4	28.0	25.3
BH 75/18-10D	Germany	Hesse	-	Fox	liver	26.5	26.1	23.4
BH 75/18-15D	Germany	Hesse	-	Fox	liver	27.2	26.2	24.2
BH 75/18-12D	Germany	Hesse	-	Fox	liver	35.8	36.7	34.3
BH 75/18-1E	Germany	Hesse	-	Fox	lymph node	20.3	21.6	18.3
BH 75/18-2E	Germany	Hesse	-	Fox	lymph node	29.9	31.1	28.2
BH 75/18-9E	Germany	Hesse	-	fox	lymph node	17.0	17.7	14.6
BH 75/18-14E	Germany	Hesse	-	fox	lymph node	21.6	22.8	20.3
BH 75/18-10E	Germany	Hesse	-	fox	lymph node	19.8	19.1	16.1

BH 75/18-15E	Germany	Hesse	-	fox	lymph node	18.6	18.5	15.0
BH 75/18-12E	Germany	Hesse	-	fox	lymph node	31.0	32.0	29.4
BH 75/18-1F	Germany	Hesse	-	fox	kidney	27.9	28.3	26.8
BH 75/18-2F	Germany	Hesse	-	fox	kidney	36.1	36.0	36.1
BH 75/18-9F	Germany	Hesse	-	fox	kidney	24.4	25.2	23.0
BH 75/18-14F	Germany	Hesse	-	fox	kidney	28.3	29.7	27.9
BH 75/18-10F	Germany	Hesse	-	fox	kidney	25.2	24.3	22.6
BH 75/18-15F	Germany	Hesse	-	fox	kidney	24.4	25.0	22.8
BH 75/18-12F	Germany	Hesse	-	fox	kidney	31.3	32.9	31.6
BH 24/18-4	Germany	Thuringia	2014	red fox	spleen	30.1	29.0	27.8
BH 75/18-3A	Germany	Hesse	-	raccoon	homogenate	31.9	31.4	33.4
BH 75/18-6A	Germany	Hesse	-	raccoon	homogenate	31.5	32.1	32.0
BH 75/18-8A	Germany	Hesse	-	raccoon	homogenate	25.0	25.0	23.5
BH 75/18-11A	Germany	Hesse	-	raccoon	homogenate	28.2	28.5	28.3
BH 75/18-13A	Germany	Hesse	-	raccoon	homogenate	26.3	27.7	27.5
BH 24/18-5	Germany	Hesse	-	raccoon	organ grinding	24.2	23.1	21.5
BH 75/18-6B	Germany	Hesse	-	raccoon	brain	26.8	27.5	26.4
BH 75/18-8B	Germany	Hesse	-	raccoon	brain	23.9	24.0	22.3
BH 75/18-11B	Germany	Hesse	-	raccoon	brain	23.2	22.6	22.9
BH 75/18-13B	Germany	Hesse	-	raccoon	brain	23.0	23.1	22.1
BH 75/18-6C	Germany	Hesse	-	raccoon	lung	34.8	34.9	33.8
BH 75/18-8C	Germany	Hesse	-	raccoon	lung	21.0	21.3	19.4
BH 75/18-11C	Germany	Hesse	-	raccoon	lung	26.0	26.1	24.5
BH 75/18-13C	Germany	Hesse	-	raccoon	lung	31.3	32.7	31.1
BH 75/18-6D	Germany	Hesse	-	raccoon	liver	32.9	33.5	31.9
BH 75/18-8D	Germany	Hesse	-	raccoon	liver	25.2	25.2	22.9
BH 75/18-11D	Germany	Hesse	-	raccoon	liver	28.8	29.5	28.1
BH 75/18-13D	Germany	Hesse	-	raccoon	liver	38.4	37.7	38.1
BH 75/18-6E	Germany	Hesse	-	raccoon	lymph node	38.2	38.3	37.3
BH 75/18-8E	Germany	Hesse	-	raccoon	lymph node	22.6	23.1	20.7
BH 75/18-11E	Germany	Hesse	-	raccoon	lymph node	24.8	25.6	22.6
BH 75/18-13E	Germany	Hesse	-	raccoon	lymph node	34.8	36.5	33.8
BH 75/18-6F	Germany	Hesse	-	raccoon	kidney	37.1	37.3	37.7
BH 75/18-8F	Germany	Hesse	-	raccoon	kidney	21.0	21.2	19.4
BH 75/18-11F	Germany	Hesse	-	raccoon	kidney	28.2	28.9	27.4
BH 75/18-13F	Germany	Hesse	-	raccoon	kidney	33.5	34.8	33.1
BH 24/18-12	Germany	Brandenburg	2007	stone marten	organ grinding	32.6	31.0	35.3
BH 24/18-11	Germany	Brandenburg	2003	dog	brain	27.5	26.8	26.3
BH 65/18-2	Austria	-	2018	fox	organ pool	32.0	32.1	31.1
BH 65/18-4	Austria	-	2018	Fox	organ pool	29.8	30.2	28.6
BH 65/18-5	Austria	-	2018	Fox	brain	28.7	28.3	27.2
BH 24/18-7	Austria	-	2008	Fox	organ grinding	29.7	28.0	27.5

BH 24/18-8	Austria	-	2008	Fox	organ grinding	26.4	23.9	22.9
BH 24/18-9	Austria	-	2010	marten	organ grinding	27.8	27.1	24.2
BH 36/19-2	Italy	-	-	-	-	15.5	16.7	14.8
BH 24/18-10	Romania	-	2016	Dog	organ grinding	30.0	28.3	28.1
BH 36/19-3	Spain	-	-	-	-	32.0	31.9	30.4
BH 36/19-1	Urkraine	-	-	-	-	13.4	15.0	13.0
BH 65/18-6	Africa	-	2018	Dog	FFPE	No Cq	No Cq	36.9
BH 105/15-1	-	-	-	-	-	21.0	18.2	19.4
BH 105/15-2	-	-	-	-	-	11.7	11.0	12.3
BH 04/18-2	-	-	-	-	-	17.1	15.8	17.4
BH 24/18-3	-	-	1995	-	-	22.2	20.8	22.5
BH 76/18-14	-	-	2012	red fox	organ grinding	No Cq	No Cq	37.5
BH 76/18-11	-	-	2012	red fox	organ grinding	33.6	34.1	34.9
BH 76/18-13	-	-	2012	red fox	organ grinding	25.3	25.1	25.3
BH 76/18-41	-	-	2013	red fox	organ grinding	21.5	22.4	20.5
BH 76/18-7	-	-	2013	red fox	organ grinding	25.0	25.5	23.4
BH 76/18-22	-	-	2013	red fox	organ grinding	35.3	35.1	36.4
BH 76/18-6	-	-	2013	red fox	organ grinding	23.0	23.8	21.4
BH 76/18-10	-	-	2012	red fox	organ grinding	37.3	36.7	34.8
BH 76/18-23	-	-	2013	red fox	organ grinding	33.5	34.8	33.4
BH 76/18-38	-	-	2013	red fox	organ grinding	30.0	30.1	29.1
BH 76/18-28	-	-	2013	red fox	organ grinding	32.4	32.6	32.6
BH 76/18-25	-	-	2013	red fox	organ grinding	32.6	32.9	33.0
BH 76/18-29	-	-	2013	red fox	organ grinding	28.9	28.9	29.1
BH 76/18-47	-	-	2013	red fox	organ grinding	29.2	29.2	29.0
BH 76/18-73	-	-	2016	red fox	organ grinding	25.8	25.8	25.2
BH 76/18-83	-	-	2016	red fox	organ grinding	30.2	31.6	32.1
BH 76/18-84	-	-	2016	red fox	organ grinding	26.2	28.5	27.8
BH 76/18-87	-	-	2016	red fox	organ grinding	24.5	25.1	24.3
BH 76/18-88	-	-	2016	red fox	organ grinding	30.6	31.8	30.1
**BH 76/18-93**	**-**	**-**	**2016**	**red fox**	**organ grinding**	**23.9**	**28.3**	**31.6**
**BH 76/18-94**	**-**	**-**	**2016**	**red fox**	**organ grinding**	**21.1**	**24.7**	**29.6**
BH 76/18-82	-	-	2016	red fox	organ grinding	25.5	26.2	24.5
BH 76/18-2	-	-	2007	red fox	brain	27.1	27.0	27.2
BH 76/18-3	-	-	2008	red fox	brain	24.4	24.1	25.9
BH 76/18-4	-	-	2009	red fox	brain	24.4	24.1	24.8
BH 76/18-21	-	-	2012	red fox	brain	27.4	26.7	27.5
BH 76/18-15	-	-	2012	red fox	brain	28.3	27.9	29.0
BH 76/18-9	-	-	2013	red fox	brain	26.7	26.7	26.5
BH 76/18-24	-	-	2013	red fox	brain	25.2	25.6	25.6
BH 76/18-55	-	-	2013	red fox	brain	27.0	27.6	27.9
BH 76/18-61	-	-	2013	red fox	brain	24.6	24.4	24.5

BH 76/18-126	-	-	2018	red fox	brain	32.8	33.7	33.8
BH 76/18-127	-	-	2018	red fox	brain	35.2	36.8	35.3
BH 76/18-136	-	-	2018	red fox	brain	34.2	35.2	34.8
BH 76/18-142	-	-	2018	red fox	brain	26.4	27.4	26.7
BH 76/18-143	-	-	2018	red fox	brain	30.4	30.8	31.1
BH 76/18-158	-	-	2018	red fox	brain	31.5	32.4	32.5
BH 76/18-161	-	-	2018	red fox	brain	22.8	22.3	23.1
BH 76/18-162	-	-	2018	red fox	brain	37.1	38.5	36.8
BH 76/18-184	-	-	2018	red fox	brain	36.7	36.8	35.8
BH 76/18-210	-	-	2018	red fox	brain	37.4	38.3	37.7
BH 76/18-90B	-	-	2016	red fox	brain	33.1	33.4	32.7
BH 76/18-91B	-	-	2016	red fox	brain	29.0	29.2	28.4
BH 76/18-92B	-	-	2016	red fox	brain	26.5	27.1	25.2
BH 76/18-93B	-	-	2016	red fox	brain	25.0	25.1	23.6
BH 76/18-94B	-	-	2016	red fox	brain	25.2	25.3	24.1
BH 76/18-97B	-	-	2008	red fox	brain	21.2	21.1	23.3
BH 76/18-98B	-	-	2008	red fox	brain	22.4	23.1	25.8
BH 76/18-75	-	-	2016	red fox	lung	22.1	23.1	20.3
BH 76/18-103	-	-	2018	red fox	lung	23.0	24.4	23.2
BH 76/18-77	-	-	2016	red fox	spleen	22.2	23.3	21.3
BH 76/18-78	-	-	2016	red fox	spleen	21.7	22.4	20.2
BH 76/18-79	-	-	2016	red fox	spleen	22.0	23.2	21.0
BH 76/18-80	-	-	2016	red fox	spleen	22.1	23.0	21.9
BH 76/18-18	-	-	2012	raccoon	organ grinding	20.8	21.5	25.3
BH 76/18-17	-	-	2012	raccoon	organ grinding	19.6	20.6	19.0
BH 76/18-5	-	-	2013	raccoon	organ grinding	16.8	17.2	15.4
BH 76/18-44	-	-	2013	raccoon	organ grinding	21.2	22.2	20.3
BH 76/18-45	-	-	2013	raccoon	organ grinding	18.6	20.0	17.7
BH 76/18-46	-	-	2013	raccoon	organ grinding	22.1	24.0	21.1
BH 76/18-48	-	-	2013	raccoon	organ grinding	24.2	24.3	23.9
BH 76/18-49	-	-	2013	raccoon	organ grinding	26.8	27.4	26.1
BH 76/18-50	-	-	2013	raccoon	organ grinding	32.1	32.2	31.6
BH 76/18-56	-	-	2013	raccoon	organ grinding	17.0	18.0	15.9
BH 76/18-52	-	-	2013	raccoon	organ grinding	20.7	21.1	19.8
BH 76/18-53	-	-	2013	raccoon	organ grinding	22.7	23.7	22.4
BH 76/18-54	-	-	2013	raccoon	organ grinding	21.8	22.3	21.1
BH 76/18-57	-	-	2013	raccoon	organ grinding	19.1	21.0	18.9
BH 76/18-58	-	-	2013	raccoon	organ grinding	20.2	21.1	18.7
BH 76/18-59	-	-	2013	raccoon	organ grinding	18.3	19.3	17.4
BH 76/18-60	-	-	2013	raccoon	organ grinding	21.2	22.0	19.4
BH 76/18-62	-	-	2013	raccoon	organ grinding	20.2	20.6	18.5
BH 76/18-63	-	-	2013	raccoon	organ grinding	17.8	18.7	21.5

BH 76/18-40	-	-	2013	raccoon	organ grinding	24.6	26.4	23.5
BH 76/18-36	-	-	2013	raccoon	organ grinding	25.9	27.7	24.9
BH 76/18-31	-	-	2013	raccoon	organ grinding	19.4	20.6	18.8
BH 76/18-32	-	-	2013	raccoon	organ grinding	21.1	21.8	20.4
BH 76/18-33	-	-	2013	raccoon	organ grinding	19.6	20.0	17.6
BH 76/18-34	-	-	2013	raccoon	organ grinding	18.5	20.0	16.7
BH 76/18-35	-	-	2013	raccoon	organ grinding	21.1	21.6	20.3
BH 76/18-26	-	-	2013	raccoon	organ grinding	21.0	22.1	19.9
BH 76/18-27	-	-	2013	raccoon	organ grinding	21.1	21.3	19.5
BH 76/18-39	-	-	2013	raccoon	organ grinding	21.6	23.0	20.7
BH 76/18-85	-	-	2018	raccoon	organ grinding	28.1	27.9	25.5
BH 76/18-86	-	-	2016	raccoon	organ grinding	22.6	24.7	21.7
BH 76/18-89	-	-	2016	raccoon	organ grinding	24.9	25.3	24.4
BH 76/18-96	-	-	2016	raccoon	organ grinding	28.7	29.0	28.4
BH 76/18-144	-	-	2018	raccoon	brain	32.5	33.8	33.2
BH 76/18-154	-	-	2018	raccoon	brain	38.6	37.7	39.3
BH 76/18-204	-	-	2018	raccoon	brain	37.5	36.5	37.0
BH 76/18-206	-	-	2018	raccoon	brain	37.3	38.4	36.8
BH 76/18-19	-	-	2012	raccoon dog	brain	26.8	27.2	27.0
BH 76/18-51	-	-	2013	raccoon dog	brain	24.0	24.0	24.9
BH 76/18-12	-	-	2012	raccoon dog	organ grinding	25.0	25.0	24.6
BH 76/18-20	-	-	2012	badger	organ grinding	26.3	26.6	25.9
BH 76/18-68	-	-	2013	badger	organ grinding	26.2	26.5	25.5
BH 76/18-100	-	-	2010	badger	organ grinding	25.6	27.3	25.1
BH 76/18-101	-	-	2010	badger	organ grinding	26.4	28.5	26.0
BH 76/18-42	-	-	2013	Wolf	organ grinding	34.5	34.7	36.7
BH 76/18-43	-	-	2013	Wolf	organ grinding	32.5	34.4	32.6
BH 76/18-67	-	-	2013	marten	organ grinding	23.2	24.3	22.3
BH 76/18-99	-	-	2010	marten	organ grinding	24.8	26.5	23.6
BH 76/18-102	-	-	2010	marten	organ grinding	19.3	22.1	19.1
BH 76/18-37	-	-	2013	marten	brain	21.5	22.0	22.1
BH 76/18-64	-	-	2013	dog	organ grinding	37.6	35.4	35.8

RT-qPCR = reverse transcriptase quantitative polymerase chain reaction, CDV = canine distemper virus, ID = ident number; Cq = quantification cycler; FFPE = formalin-fixed paraffin-embedded; * year of isolation
